# Semantic mechanisms may be responsible for developing synesthesia

**DOI:** 10.3389/fnhum.2014.00509

**Published:** 2014-08-19

**Authors:** Aleksandra Mroczko-Wąsowicz, Danko Nikolić

**Affiliations:** ^1^Institute of Philosophy of Mind and Cognition, National Yang-Ming UniversityTaipei, Taiwan; ^2^Ernst Strüngmann Institute (ESI) for Neuroscience in Cooperation with Max Planck SocietyFrankfurt, Germany; ^3^Frankfurt Institute for Advanced StudiesFrankfurt, Germany; ^4^Department of Neurophysiology, Max Planck Institute for Brain ResearchFrankfurt, Germany; ^5^Department of Psychology, Faculty of Humanities and Social Sciences, University of ZagrebZagreb, Croatia

**Keywords:** synesthesia, perception, cognition, concepts, learning, semantics, cognitive penetrability

## Abstract

Currently, little is known about how synesthesia develops and which aspects of synesthesia can be acquired through a learning process. We review the increasing evidence for the role of semantic representations in the induction of synesthesia, and argue for the thesis that synesthetic abilities are developed and modified by semantic mechanisms. That is, in certain people semantic mechanisms associate concepts with perception-like experiences—and this association occurs in an extraordinary way. This phenomenon can be referred to as “higher” synesthesia or *ideasthesia*. The present analysis suggests that synesthesia develops during childhood and is being enriched further throughout the synesthetes’ lifetime; for example, the already existing concurrents may be adopted by novel inducers or new concurrents may be formed. For a deeper understanding of the origin and nature of synesthesia we propose to focus future research on two aspects: (i) the similarities between synesthesia and ordinary phenomenal experiences based on concepts; and (ii) the tight entanglement of perception, cognition and the conceptualization of the world. Importantly, an explanation of how biological systems get to generate experiences, synesthetic or not, may have to involve an explanation of how semantic networks are formed in general and what their role is in the ability to be aware of the surrounding world.

## Introduction

Following a long history of interdisciplinary research on synesthesia (see e.g., Locke, 1689; Galton, 1883/1907/1973; Mahling, 1926; Peacock, 1988; Cytowic, 1995, 2002; van Campen, 1999; Day, 2005; Dixon and Smilek, 2005; Jewanski and Sidler, 2006; Macpherson, 2007; Brogaard, 2013; Auvray and Deroy, 2014), at the beginning of the 21st century an important debate has emerged about the fundamental nature of synesthesia. Initially, the discussion was dominated by the explanation of synesthesia in terms of intermixed senses. It was presumed that a sensory input in one modality directly elicits an additional sensory activation in another sensory modality, and this was further deduced to be based on direct connections between the respective brain areas, known also as the cross-activation hypothesis (e.g., Baron-Cohen and Harrison, 1997; Ramachandran and Hubbard, 2001a; Cytowic, 2002).

However, meanwhile, this interpretation has encountered problems. It has become apparent that in many forms of synesthetic associations, only the concurrents exhibit clear sensory-like properties, while the inducers take the form of concepts. For example, one of the most common forms of synesthesia is time-unit synesthesia (Smilek et al., 2007a; Jarick et al., 2008, 2009, 2011; Brang et al., 2013b) in which weekdays or months are associated with colors, distinct spatial positions and with other concurrents. Importantly, there are no direct sensory inputs for time units. One cannot directly see June or Thursday in a way one can see a car or letter “A”. Time units exclusively exist as concepts. But time-unit synesthesia is not the only case. For other forms of synesthesia the traditional, sensory-to-sensory characterization has also been found unsatisfactory (Ward and Simner, 2003; Ward, 2004; Simner, 2007; Nikolić et al., 2011; Mroczko-Wąsowicz and Werning, 2012). Thus, it has been repeatedly suggested that a full account of the phenomenon should go beyond the standard sensory-sensory approach (e.g., Dixon et al., 2000, 2006; Simner and Ward, 2006; Ward et al., 2006; Ward and Sagiv, 2007; Sagiv et al., 2011; Eagleman, 2012; Jürgens and Nikolić, 2012, 2014; Simner, 2012; Watson et al., 2012a, 2014; Mroczko-Wąsowicz and Nikolić, 2013; Chiou and Rich, 2014; Brogaard, 2014; Brogaard et al., 2014).

A number of different remedies to the problem of an adequate characterization of synesthesia have been proposed. An attempt to bridge this discrepancy between the theory and the data was to postulate individual differences among synesthetes (Dixon and Smilek, 2005; Hubbard et al., 2005; Cytowic and Eagleman, 2009; Rouw and Scholte, 2010; Rouw, 2011; Simner, 2013). Thus, there would be two main groups, higher and lower synesthetes, depending on whether their synesthesia is triggered by the conceptual or by the sensory properties of a stimulus, respectively (Ramachandran and Hubbard, 2001b). Another approach was to suggest that the phenomenon oscillates between these two types of inducers within one and the same individual (Hubbard et al., 2005; Ward et al., 2007).

It can be said that the last decade experienced a renewed interest in re-conceptualizing and re-defining the phenomenon of synesthesia (Simner, 2007, 2012; Sagiv et al., 2011; Cohen Kadosh and Terhune, 2012; Mroczko-Wąsowicz and Werning, 2012; Jürgens and Nikolić, 2014). Researchers have started to question the exclusively sensory interpretation of synesthesia and have suggested that the phenomenon also involved high-level cognitive representations (Ward et al., 2006, 2007; Ward and Sagiv, 2007). This includes the proposal that synesthesia is exclusively a semantically induced phenomenon (Nikolić, 2009; Chiou and Rich, 2014). This suggestion severs the traditional distinction between perception and cognition assumed for a long time in philosophy, psychology and cognitive science (Mroczko-Wąsowicz and Werning, 2012; Mroczko-Wąsowicz, 2013). Evidence for this alternative, extended view of synesthesia is continually increasing (Dixon et al., 2000, 2006; Grossenbacher and Lovelace, 2001; Elias et al., 2003; Mroczko et al., 2009; Nikolić et al., 2011; Simner, 2012; Rothen et al., 2013; Jürgens and Nikolić, 2014). The aim of the present paper is to examine these fundamental issues and the particular forms of the phenomenon for which the hypothesis applies that semantic mechanisms create synesthesia.

## Mind-driven higher synesthesia

In synesthesia, an attended and recognized stimulus leads to additional phenomenal experiences, that is, to experiential states that normally do not occur (Rich and Mattingley, 2003; Mattingley, 2009; Nijboer and Van der Stigchel, 2009; Mroczko-Wąsowicz and Nikolić, 2013). As a result, the stimuli corresponding to the inducer and the experiences associated with the concurrent form a highly integrated percept—a phenomenally unified experience (Mroczko-Wąsowicz and Werning, 2012; Mroczko-Wąsowicz, 2013). This unified experience may cover the same sensory modality, or different sensory modalities, or even different domains e.g., cognitive, sensory and motor. The resulting conscious experiences are typically unified, meaning that all the experiences at a given time are present simultaneously forming an overall phenomenal perspective—a single encompassing phenomenal state. The different component experiences are said to be co-conscious parts or aspects of the subsuming state that has a conjoint phenomenology, or a joint phenomenal content (Shoemaker, 1996, 2003; Bayne and Chalmers, 2003; Tye, 2003; Brook and Raymont, 2013). As such, they are intimately linked and integrated from the perspective of the self. Consequently, a person’s perceptual and cognitive states result in a synchronic phenomenal unity (Raymont and Brook, 2009; Bayne, 2010; Mroczko-Wąsowicz, 2013).

One of the most important issues in the current debate about the phenomenon of synesthesia pertains to the putative causal mechanisms underlying synesthetic associations. The question of whether synesthesia is a lower or higher phenomenon translates into a question of whether novel synesthetic associations can be explained by direct synaptic connections between neurons representing respectively the inducer and the concurrent—which is a possible explanation of the low-level hypothesis. Alternatively, synesthetic associations may occur through a high-level of system organization, that enables associations to be formed in a more elaborated, distributed and flexible manner than what can be accounted for by direct synaptic connections between sensory areas. The latter would correspond to the high-level hypothesis of synesthesia and is closer to the level at which the semantic structure of knowledge operates: rich association systems involving additional brain areas as opposed to direct connections between two sensory areas of the brain (for a recent hypothesis on the involvement of ventral IT-cortex see Chiou and Rich, 2014). At this higher level a prominent role is played by context, attentional mechanisms, and interpretation of the stimuli.

The two levels are often referred to as *perceptual* and *conceptual* (or *cognitive*), respectively (Ward and Simner, 2003; Eagleman, 2012; Simner, 2012; see also Evans, 1982). This is because it is typically assumed that perception is an earlier and hence, more rudimentary stage of information processing than is the activation and processing of semantic information. Nevertheless, the distinction between higher and lower synesthetes responding to respectively, high- and low-level features of inducing stimulus has rarely been a topic of direct investigation.

Many forms of synesthesia indicate the involvement of semantic processes i.e., of the meaning of the stimulus. Synesthetes often associate the same concurrent (e.g., the same hue of a color) with different physical representations of the stimulus, i.e., with different sensory instantiations of the same concept (e.g., the Arabic digit “4”, the Roman numeral “IV”, the word “four”, and four dots on a dice; Ramachandran and Hubbard, 2001b; Ward et al., 2007). Number-color synesthesia can be induced via sensory presentation (e.g., digits), but also conceptually via cardinality (the quantity) or via another conceptual property of ordinality (the position in a sequence).

There are two other common forms of synesthesia that can be mind-driven and that are variants of the already mentioned time-unit synesthesia: the first is known as *colored sequence synesthesia* in which names of time units, such as days of the week and months of the year, are colored differently from their graphemic constituents (Simner et al., 2006; Tomson et al., 2013), and the second is known as *spatial sequence synesthesia* in which ordinal categories involving numbers, time and alphabet elicit experience of spatial forms (Sagiv et al., 2006; Eagleman, 2009).

A related phenomenon observed in non-synesthetes, which suggests a bridge between synesthesia and non-synesthetic perception, is the spatial numerical association of response codes. Here, overlearned ordinal stimuli are associated with implicit spatial representations (Dehaene et al., 1993).

Some neuroanatomical studies have suggested that differences between higher and lower synesthetes map onto another, more empirically grounded, distinction between associators and projectors (Ramachandran and Hubbard, 2001a,b; Dixon et al., 2004; Dixon and Smilek, 2005; Hubbard et al., 2005; Rouw and Scholte, 2010; van Leeuwen et al., 2011). The assumption is that a semantic representation of number (relevant for higher synesthetes) is likely linked to a more conceptual representation of color (relevant for associators experiencing their colors internally in their “mind’s eye”). However, recent evidence seems to challenge this view suggesting that the two distinctions are not the same but rather orthogonal (Ward et al., 2007). Ward and colleagues propose that the mechanisms that give rise to the associator-projector distinction are independent of those that give rise to higher-lower characteristics. According to the researchers, the former reflects an internal vs. external frame of spatial reference related to synesthetic phenomenology, and the latter reflects the extent to which conceptual vs. sensory properties of the stimulus are involved in the induction of synesthesia. Along with the results of the behavioral tests and subjects’ phenomenological reports, they found that higher synesthetes were not necessarily grapheme-color associators, but could also be projectors.

As an explanation of the finding Ward et al. (2007) proposed to differentiate between surface-projectors and near space-projectors. Surface projectors evoke an externalized frame of reference relative to the location of the stimulus, perceiving their synesthetic color concurrents as located in the same spatial location as the inducing graphemes, dice patterns, finger counting, etc. In contrast, near space-projectors elicit an externalized frame of reference relative to the location of their body. For example, the concurrent may always be located 20 cm in front of the perceiver. This dissociation may help explain varying results for projectors. Projectors are shown to be faster in naming their synesthetic colors than veridical colors, and associators exhibit the opposite effect (Smilek et al., 2001, 2003). However, projectors who project colors into external space but not onto the surface of graphemes may behave more like associators on the task of naming their concurrents. Near space-projectors may be slower with synesthetic colors than with ink colors as a result of the need to shift their attention from the grapheme location to the color location. In contrast, surface-projectors place concurrents at the same location as the attended stimuli, which may then enable more efficient naming of synesthetic colors.

The Ward et al. (2007) study also suggests that it may well be possible for a synesthete to demonstrate higher synesthesia in one respect but lower in another, e.g., experiencing number forms may be shared by two groups, associators and projectors. The respective internal and external location of spatial forms does not have to reflect the spatial reference of their experiences when, for example, reading graphemes. One and the same synesthete may be an associator in his number forms but a projector for his grapheme-colors. In line with this, a comparable oscillation between high and low synesthesia may occur within the same person for the different forms of synesthetic associations the person possesses.

This view is supported by studies on numerical cognition (Cohen Kadosh et al., 2005; Cohen Kadosh and Henik, 2006a,b) in which digit-color projectors showed a characteristic feature of higher synesthesia when linking colors with the meaning of numbers or with information on magnitude. Similarly, both projectors and associators showed Stroop-like effects when perceiving color for an arithmetic sum in which the result was implied and not visually presented and hence, had to be extracted through semantic mechanisms (Dixon et al., 2000; Jansari et al., 2006). For lower synesthetes, color would have to be linked to the visually presented physical form of the digit, and not to the conceptual aspects of a number. Hence, it is possible that for different individuals the semantic structures that lead to synesthesia are organized differently. Some synesthetes may be higher in the sense of visuo-spatial properties such as the number forms and ordinality (and spatial forms for time and the alphabet since these concepts also possess ordinality), while other synesthetes may be higher in the sense of numerosity (cardinal aspects of number meaning, number concepts). And yet, there could be a group that combines the two effects, which then results in having the same color for “January” and “1”, “February” and “2”, etc. (Sagiv et al., 2006).

Examples of cases of synesthetic experiences arising in the absence of any direct impact or physical presence of the stimulus, or occurring independently of the various physical forms that external inducers may take, are mind-dependent or mind-driven higher synesthesia, also known as ideasthesia. We suggest that these experiences are driven by semantic mechanisms as a part of mental representations whereby each synesthete’s individual semantic network contains concurrents as a part of the meaning of the inducing stimuli (Jürgens and Nikolić, 2014). A similar proposal for a conceptual contribution to synesthesia was made by Meier (2013). Meier’s approach is based on the research on implicit bidirectionality (Brugger et al., 2004; Johnson et al., 2007; Weiss et al., 2009), and states that processing of an inducer is affected by a spreading of implicit activation of a concurrent. This is in agreement with our claim that synesthetic associations include unconventionally coupled supplementary phenomenal features as a part of the semantic knowledge network. Therefore, synesthetic experiences do not have to rely on external stimuli to be induced, i.e., cognitive or concept-dependent mental states are reported to be sufficient to elicit synesthetic perceptual concurrents in the absence of any related physical stimulation (Nikolić et al., 2011; Mroczko-Wąsowicz and Werning, 2012).

**Figure 1:**
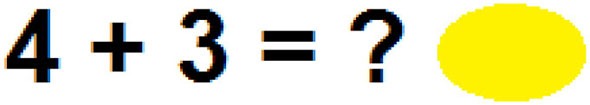
**An example of the stimuli used in the experiment of Dixon et al., 2000)**.

The above-mentioned case of a digit-color synesthete studied by Dixon et al. (2000) using mental arithmetic, provided a striking example of conceptual synesthesia. Not only viewing digits triggered a visual experience of a specific color. Also a mental activation of the concept of the calculated number was fully sufficient to evoke synesthesia (e.g., even without writing down the result of the calculation) (Figure 1). This was revealed when the synesthete performed mathematical additions such as “4 + 3” followed by a patch colored congruently or incongruently to the synesthetic color associated with the result of the summation (e.g., yellow for digit 7). The result of summation was never directly presented. The synesthete had to name the color of the patch before reporting the result of the summation. The responses were slower in the case of incongruency i.e., when the color of the patch was different from the color associated with the arithmetical sum. This Stroop-like test objectified the mind-driven case of synesthesia demonstrating that the cause of this synesthetic experience is not provided by the immediate sensory stimulation.

Another source of evidence of mind-dependent synesthesia has been provided in studies with letter-color synesthetes reporting to experience the same concurrent to different physical stimuli representing the same *type*, *kind* or *semantic category*. Experiences were the same for letters presented in different alphabets (Mroczko et al., 2009), various fonts, or presented via different sensory modalities—visual or auditory (Simner, 2007). Similarly, number-color synesthetes show indifference to a wide range of physical instantiations of numerosity (Dixon et al., 2000; Ward and Sagiv, 2007).

Such sets of low-level sensory features or objects identified as members of a familiar meaningful category are the subject of recent philosophical discussions about object recognition and the related high-level vs. low-level content of perception referring to a *kind* property (i.e., when recognizing that something belongs to a certain kind or type, like being a tiger, being a pine tree, etc.) (Pylyshyn, 1999; Siegel, 2006; Bayne, 2009). The same analysis might apply to typical synesthetic inducers such as seeing letters “A”, “𝔸” and “a” all *as “*A”, that is, recognizing an exemplar of a certain semantic category (i.e., a token of a type). There is no consensus on how to interpret such content, as high-level conceptual or low-level perceptual, but an agreement is that this content is cognitive, at least in some general sense.

**Figure 2:**
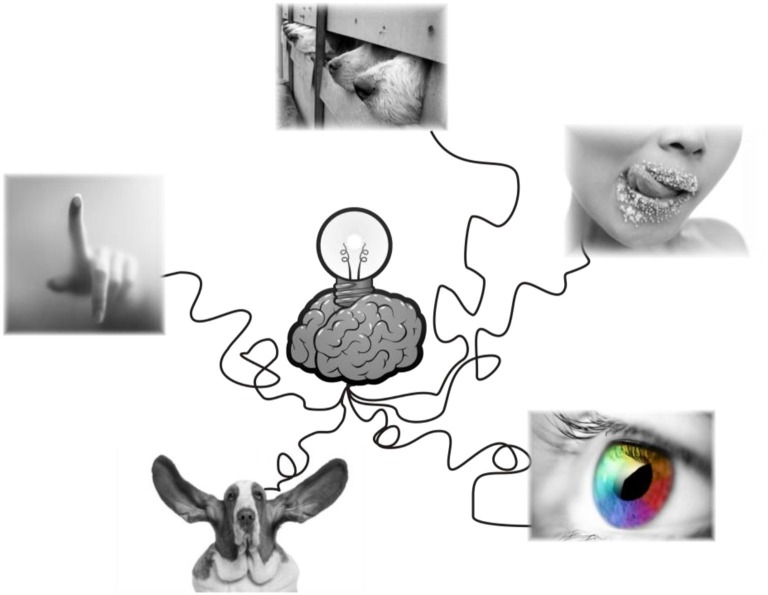
**Illustration of the view that conceptual contents might be the inducers of idiosyncratic synesthetic experiences**.

A related issue is that of the mode of recognition that applies to such a kind property (Simner, 2007; Auvray and Deroy, 2014; see Pylyshyn, 1999 for the argument that a computation based on perceptual principles inherent to categorical perception may lead to low-level detection of similarities between stimuli). We propose that at least for some forms of developmental synesthesia, if not for all, the concurrent is activated only after semantic decoding of the inducer is completed—i.e., the meaning of inducer must be extracted before concurrent can be experienced. As mentioned, a number of studies provide convincing evidence for the role of conceptual contents in the induction of synesthesia (see Figure 2 for an illustration of that proposal).

Thus, the studies confirm that synesthetic experiences are largely internally-driven by mental representations of objects, higher-order types and semantic categories, cognitive states and events, or other internal determinants such as concepts, thoughts, moods, memories and imagery (Spiller and Jansari, 2008; Rothen et al., 2012; Meier and Rothen, 2013; Simner, 2013; Ward et al., 2013). Another form of higher synesthesia has been observed in two different individuals who acquired their color/shape synesthesia for digits and mathematical formulas; interestingly the visual concurrents were generated in higher parietal and frontal brain regions (Bor et al., 2007; Brogaard et al., 2013). Since the cognitive representations seem to be factors sufficient to elicit synesthesia they may also play a role in explaining variations of synesthetic experiences.

In the following section, we make the point that conceptual properties are shared across many forms of synesthesia. This makes us consider higher (semantic) synesthesia a separate class, which ought to have its own place in the synesthetic taxonomy and has to be considered accordingly in the definitional and explanatory efforts of investigations into the phenomenon.

## Conceptual aspects and semantic mechanisms involved in different forms of synesthesia

To substantiate the importance of studying higher synesthesia, we highlight numerous conceptual aspects and semantic mechanisms involved in various types of synesthesia. An account directly addressing the influence of semantic information on the induction of synesthetic experience is “ideasthesia” which literally means *sensing concepts*, and refers to the conceptual processing underlying synesthesia in which only concurrents are perceptual while inducers are conceptual (Nikolić, 2009). According to this model, high-level semantic mechanisms assign low-level sensory concurrents (e.g., synesthetic colors are triggered only when a synesthete extracts the meaning of the presented grapheme; see also Jürgens and Nikolić, 2012, 2014; Milán et al., 2013; cf. Brang et al., 2013a).

Synesthetic associations are not just cross-modal, but they also cross domains, i.e., besides the various modalities of senses, these associations may involve domains of bodily, motor and emotional states as well as domains of abstract, conceptually-represented entities like numbers or time units. Cases of synesthetic inducers going beyond traditionally denoted sensory modalities have been found for activities such as reading musical notes, calculating, imagining, or just thinking of a stimulus (Frith and Paulesu, 1997; Grossenbacher, 1997; Dixon et al., 2000, 2006; Ramachandran and Hubbard, 2001b; Cytowic, 2002; Rich et al., 2005; Ward et al., 2006; Spiller and Jansari, 2008; Nikolić et al., 2011).

Studies with bilingual grapheme-color synesthetes have shown that synesthetesia can be experienced also for alphabets different from the one used in the first language (Mills et al., 2002; Rich et al., 2005; Witthoft and Winawer, 2006). In such a cross-linguistic transfer synesthetic colors from a second language are usually mapped onto the existing colors of the first language (Simner, 2007; Mroczko-Wąsowicz and Nikolić, 2013). Often, the color is associated with a letter by its similarity to other letters, i.e., letters of similar shapes tend to elicit similar colors (Brang et al., 2011a; Jürgens and Nikolić, 2012, 2014).

**Figure 3:**
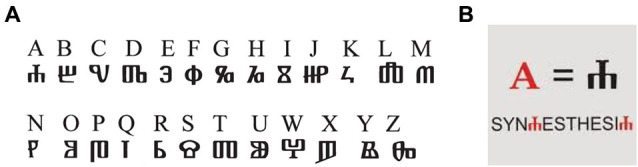
**(A)** The correspondence between the graphemes of Latin and square Glagolitic alphabets. **(B)** Illustration of the color experience associated with a new grapheme after a short training. Both figures republished with permission of ARVO, from Immediate transfer of synesthesia to a novel inducer. Mroczko et al. (2009); permission conveyed through Copyright Clearance Center, Inc.

Results from our own studies suggest that synesthetic associations to new graphemes are established quickly, and are not created from scratch but are inherited from existing associations, the concurrent colors being passively adopted from the original synesthesia (Mroczko et al., 2009; Mroczko-Wąsowicz and Nikolić, 2013). To demonstrate this, we conducted a study with 16 grapheme-color synesthetes in which we attempted to replicate in the laboratory conditions the natural phenomenon of cross-linguistic transfer. We introduced Glagolitic graphemes (novel shapes, without any corresponding sound), which come from an ancient Eastern European writing system that was ideal for our study; it had the sufficient exotic appearance. Only few letters bore any physical resemblance to other known graphemes (see Figure 3; Franolić and Žagar, 2008).

We assigned to each new grapheme a meaning by linking it to existing Latin letters and Arabic numerals. A simple writing exercise with paper and pencil sufficed to imitate the natural cross-linguistic transfer. The exercise lasted less than 10 min during which subjects learned how to write a Glagolitic grapheme and then used this knowledge to write 20 words or number sequences. The subjects substituted one familiar Latin/Arabic grapheme with its Glagolitic counterpart (as in Figure 3B). This resulted in a quick transfer of the original synesthetic color experiences from their native Latin alphabet to Glagolitic graphemes never seen before the experiment (Mroczko et al., 2009). Also, Stroop-type tests (Stroop, 1935; Mills et al., 1999; Nikolić et al., 2007) indicated that these new synesthetic associations were immediate and involuntary.

The novel synesthetic associations were created within minutes, which was too rapid a process to be accounted for by low-level sensory cross-wiring between grapheme and color brain areas. Low-level perceptual learning is usually not cognitively penetrable (see below) and, as a result, it is a relatively slow process requiring typically thousands of trials (Ahissar and Hochstein, 1997; Goldstone, 1998; Seitz and Watanabe, 2005). In contrast, the meaning of a new symbol can be learned quickly (often in a single trial; Markson and Bloom, 1997; Bloom, 2000). Thus, shorter transfer times of synesthetic associations are expected if the associations originate at the semantic level of representation. In other words, the speed of transfer is indicative of the type of representation (high- vs. low-level).

Additionally, the new associations generalized to the exemplars of different physical representation (handwritten by another person), which is consistent with learning a semantic category of stimuli rather than individual exemplars, or particular motor coordinates, and is also inconsistent with perceptual learning. The ways in which these stimuli were similar can be described only as semantic “similarities”, i.e., they shared the same meaning and not elementary visual features presumably processed in the grapheme area (Brang et al., 2011a).

Therefore, a better explanation of grapheme-color synesthesia can be provided by high-level conceptual mechanisms than by low-level associations: arbitrary synesthetic colors can be assigned to novel graphemes through transfer of semantic knowledge. This suggests the involvement of the high-level stimulus properties in eliciting synesthetic colors. The newly created synesthetic associations to Glagolitic letters must have been induced via existing synesthetic associations previously linked to conceptual representations of the Latin letters. In other words, after training, a Glagolitic letter would activate the same semantic content that is normally activated by a Latin letter. Here, as for the other cases of higher synesthesia, the meaning of the stimulus has to be extracted before the concurrent phenomenal aspect can be included into an overall unified synesthetic experience (Edquist et al., 2006; Simner, 2007; Mroczko-Wąsowicz, 2013). Therefore, synesthesia seems to rely essentially on a certain conceptual representation of the stimulus.

The significance of meaning and context in grapheme-color synesthesia was demonstrated also in the study “Is the sky 2?”, using electroencephalography to measure event-related potentials (Brang et al., 2008). The authors concluded that connections between colors and numbers are bidirectional and that concurrent color sensations are treated by synesthetes as meaningful stimuli. The impact of semantic activation and linguistic modulation such as numerical magnitude or the frequency of grapheme use in a language may also be reflected in the saturation and luminance of the experienced colors (Beeli et al., 2007; Cohen Kadosh et al., 2007; Smilek et al., 2007b; Watson et al., 2012b; see also work by Akins et al. on different languages’ effects upon grapheme-color synesthesia). Moreover, synesthetic colors elicited by ambiguous graphemes are modified by context. They depend on the interpretation of the grapheme either as a letter or as a digit (e.g., the grapheme “V” referring to Latin letter and Roman numeral; Ramachandran and Hubbard, 2001b; Dixon et al., 2006).

Another example providing support for the view that semantic mechanisms may be responsible for developing synesthesia, is number-color synesthesia. Here, it is the higher cognitive numerical representation, the conceptual representation of a number (numerosity, quantity), that is responsible for eliciting synesthetic concurrent experiences (Butterworth, 1999; Cohen Kadosh et al., 2005; Cohen Kadosh and Henik, 2006a,b). In such a case, same colors are elicited from physically different representations of a number (e.g., Arabic and Roman numerals, arrays of dots, dice patterns, finger counting or number names), provided they match in the indicated quantity (Dixon et al., 2006; Ward and Sagiv, 2007; Ward et al., 2007).

In case of projectors, colors may be projected not only on the surface of a page when viewing written digits, but also on the surface of a dice or on synesthete’s fingers when counting. The color reported for all these different physical instantiations of number concept is the same. The cardinality is the direct cause of the sameness of color across various notational formats (Ward and Sagiv, 2007). During development, the synesthetic cardinality-color associations may become generalized to other education-based and culturally acquired symbols. Other forms of synesthesia, described above, confirm that color concurrents may transfer from one physical system of representation to another on the basis of semantic correspondences, due to shared conceptual properties (Mroczko et al., 2009; Jürgens and Nikolić, 2012). For some synesthetes the colors of the writing system from a second language are taken from the colors of the alphabet of their first language (Mills et al., 2002; Witthoft and Winawer, 2006; Mroczko et al., 2009; Mroczko-Wąsowicz and Nikolić, 2013), or the synesthetic color experience associated with a written musical note acquires the color of the name denoting it (Ward et al., 2006).

Other examples might be number-forms synesthesia, time-units or calendar synesthesia, all of which involve experiencing numbers or time-units within a space. Thinking about sequential concepts, such as numerical sequences in a form of days of the week or months, elicits spatial forms and visualized mental maps that contain arranged numbers (Sagiv et al., 2006). For example, a subject may experience units of time as being arranged in an ellipse, column or a spiral. Moreover, this spatial structure is usually placed at a specific spatial location within the three-dimensional space surrounding the body of the synesthete. Alternatively, the structure is located within a virtual space not positioned relative to the body but within the mind’s eye (e.g., Smilek et al., 2007a; Jarick et al., 2008; Mann et al., 2009; Simner et al., 2009).

Days of the week as well as months present maybe the most apparent form of a conceptual inducer, as they do not possess any characteristic sensory properties. There is nothing perceptually specific that distinguishes a Monday from a Wednesday or May from August.

Another illustrative example of how concepts have impact on synesthetic experiences is given by lexical-gustatory synesthesia. In this case, different flavors are experienced in response to various words. Interestingly, such synesthetes can perceive a gustatory sensation even before articulating a given word thus, while the word is still on the tip of their tongue i.e., in the semantic phase (Simner and Ward, 2006). This demonstrates that the physical instantiation of the stimulus (i.e., the somato-sensory experience of verbalization, the spoken expression of a thought in words) is not necessary for eliciting synesthetic sensations of a flavor. The activation of the concept, which is grasped mentally, is fully sufficient.

Higher cognitive aspects of inducing synesthesia can furthermore be identified in mirror-touch or mirror-pain synesthesia. When seeing another person being touched or being in pain, any person may trigger corresponding emotions and empathy. In synesthetes however, in addition, a respective tactile or painful experience may be produced. And this experience has the quality of real touch and pain, even being located on the equivalent part of synesthete’s body (Blakemore et al., 2005; Banissy and Ward, 2007, 2013; Fitzgibbon et al., 2010).

**Figure 4:**
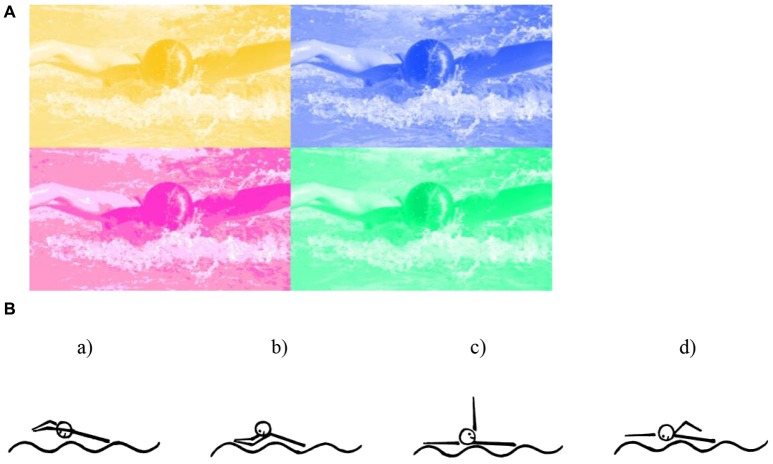
**(A)** Stimuli that can be used in the Stroop-type test: example pictures of a person performing a butterfly stroke, painted either in a subject’s synesthetic color (congruent) or in one of his non-synesthetic colors (incongruent). Reprinted from Swimming-style synesthesia. Nikolić et al. (2011) with permission from Elsevier. **(B)** Pictograms of the swimming-styles: (a) butterfly, (b) breaststroke, (c) backstroke, and (d) crawl used in Rothen et al. (2013). Reprinted from Psychophysiological evidence for the genuineness of swimming-style colour synaesthesia. Rothen et al. (2013) with permission from Elsevier.

To the list of synesthesias shown to be based on semantic interpretation of the stimulus belongs also a recently discovered swimming-style-to-color-synesthesia in which seeing another person swimming with a given swimming style, or even just thinking about a certain swimming style, elicits synesthetic color experience (Nikolić et al., 2011; Mroczko-Wąsowicz and Werning, 2012; Rothen et al., 2013) (see Figure 4).

Collectively, all these findings impose constraints on theories of the neurophysiological origins of synesthesia. Synesthetic experiences often involve inducers that are not strictly sensory (i.e., words, letters, numbers, time units, musical notes, personalities, emotions, or movement styles). Conceptual components, identified in many forms of synesthesia, transcend the traditionally believed sensory nature of inducers. Synesthesia seems to rely on a certain interpretation of the stimulus and the meaning that it has for the subject, and this meaning may change dynamically depending on the context (Rich and Mattingley, 2003; Dixon et al., 2006; Ward et al., 2006).

## Cognitive penetrability and synesthesia

These conclusions suggest that some forms of synesthesia may be understood as cases of cognitive penetrability under the conditions in which the sensory experience normally is not penetrable. Pylyshyn (1999) introduced the concept of cognitive penetrability and the lack thereof, to account for the fact that in some cases visual experience of a perceived object is fully independent of how we cognitively understand the object or what we know about that object. For example, in the Mueller-Lyer illusion one of the two lines continues to appear shorter irrespective of the direction of attention, our knowledge that the lines are equally long, or of any other form of mental effort. Thus, in this case the perceptual experience cannot, in any known way, be affected by cognition. In other words, the sensory property of visual experience is cognitively impenetrable. Pylyshyn referred to these impenetrable aspects of vision as early vision.

Synesthesia may be a case in which cognitive penetration also takes place for early sensory processing. For example, the perception of colors is usually considered impenetrable, i.e., cognitive operations cannot change the experience of the color of a perceived object (Brogaard and Gatzia, in press; but cf. Macpherson, 2012; Siegel, 2012; Vetter and Newen, 2014). At least this seems to be the case for non-synesthetes. Synesthetes are special in that they are able to penetrate this early aspect of vision (or other sensory modalities) by certain higher cognitive mechanisms. This means, synesthetic perceptual concurrents can be modified by conceptual contents, contextual expectations, linguistic modulation and cultural factors being their inducers (Dixon et al., 2000; Simner, 2007, 2012; Mroczko et al., 2009; Meier, 2013; Mroczko-Wąsowicz and Nikolić, 2013; Brogaard et al., 2014). Activating a concept synesthetes can produce a top-down influence on a sensory experience. Thus, synesthetes exhibit a specific difference from non-synesthetes in the structure of their cognition. The ubiquity of this difference is relatively small, as it applies to a small subset of the concepts that a given person possesses and uses throughout the lifetime. For example, it may apply only to certain graphemes or words. Nevertheless, the very existence of such penetrability is important for the science of mental phenomena. It tells us that the organization of the mind is more flexible than usually presumed.

## The physiology of semantic representations

Usually, one expects to assign to any mental event or faculty a brain region that is being considered its physiological underpinning. For example, if we consider associations between auditory inducers and visual concurrents, it is expected that there are distinct brain areas for each and that the physiological account solely involves those two brain areas. Similarly, if semantic representations are included in understanding the functioning of synesthesia, one automatically expects that a corresponding third brain area will be identified and will become a mediator of the flow of activation, i.e., from auditory, through semantic, to visual area.

In fact, this traditional one-area-for-function approach has already been applied to the semantic processes in synesthesia. Chiou and Rich (2014) propose that the anterior temporal lobe (ATL) is a hub for inducing synesthesia and that a transcranial magnetic stimulation (TMS) can lead to a breakdown of the coupling between inducers and concurrents. If their proposed research agenda is executed, we will probably soon know whether this part of the cortex plays a certain role in forming synesthetic associations.

However, there are reasons to believe that, irrespective of the results those future studies bring, this may not be the whole story. Semantics may be something that is not particularly localizable, but is instead to a high degree a distributed property of the brain. For example, there is evidence that the motor cortex plays an important role in the acquisition of action-related concepts (Gallese and Lakoff, 2005). Thus, the role of the motor cortex may be particularly important when a subject needs to prepare for a behavioral interaction with the object. Hence, the outcome of the question of whether semantics can be localized or whether it is a distributed function also depends on what is meant by semantics. In a generalized form, a concept may serve an extended function, not only to give a label to the stimulus, but also to prepare the person to interact with the conceptualized stimulus. If this interaction requires manual operations, the corresponding part of the motor cortex may be as important as the part of the infero-temporal cortex (Barsalou et al., 2003; Gallese and Lakoff, 2005).

Therefore, to fully account for the physiological underpinnings of the semantic mechanisms in synesthesia, we propose that future studies should not focus on a limited, pre-specified set of brain regions. Instead, a brain-wide analysis should be conducted with equal emphasis on all of its parts. In the past, such non-restrictive analyses have been tremendously useful. For example, the analysis of connectivity in grapheme-color synesthesia (Rouw and Scholte, 2007) has shown that anatomically much more changes than connections between grapheme area and color areas. Anatomical differences associated with synesthesia are wide spread across the brain. We suggest that these anatomical changes reflect the widespread nature of semantics in the cortex. That is, semantics may be a function of the entire cortex, and not a function of a spatially bound module. In other words, if we accept the premise that synesthesia is largely a semantic phenomenon, the findings by Rouw and Scholte reveal the ensuing anatomical changes of a global network. This global system may be serving the various semantic aspects of graphemes and may play a role in its entirety when synesthetic concurrents are being assigned to those graphemes.

Meanwhile, the evidence has considerably expanded that synesthesia is not a function of local cortical circuits but a global phenomenon (Hänggi et al., 2011; Hupé et al., 2012), and so did the support for the arguments that synesthesia relates to high-level cognitive functions and abilities, including memory and creativity (Rothen and Meier, 2010; Rothen et al., 2012; Mulvenna, 2013; Meier, 2013). One implication is that the research on semantics in the brain is itself incomplete. Thus, synesthesia research does not have the luxury of building on well-established physiological underpinnings of semantics. Rather, synesthesia research has to coevolve with the research on semantics, helping us understand better semantics of natural language and cognition—a feat critically important for cognitive science. Therefore, in future studies, research on how our brain processes meanings may evolve hand-to-hand with research on synesthesia.

In this effort, the recently proposed theory of the organization of adaptive system, named practopoiesis, may provide valuable guidance (Nikolić, 2014). According to this theory, semantic mechanisms are closely related to the mechanisms of general knowledge stored in long-term memory on the basis of which more specific knowledge is extracted, which is stored in working memory. The theory suggests that not only phenomenal experiences in synesthesia, but also everyday experiences, may be related to the process of categorization—synesthesia being a useful trait to enhance this process (see also Jürgens and Nikolić, 2014). Synesthesia may thus develop in childhood to assist activation and manipulation of particularly abstract semantic contents.

These conclusions lead to a very different set of hypotheses that can be formulated about synesthesia than the traditional hypotheses on brain mechanisms known as cross-activation hypothesis (i.e., based on neuronal excitation) (Ramachandran and Hubbard, 2001a; Hubbard et al., 2005) and its alternative, disinhibition hypothesis (Armel and Ramachandran, 1999; Grossenbacher and Lovelace, 2001). We discuss those novel predictions next.

## Development of synesthesia

In case that synesthesia is a phenomenon the induction of which is predominantly semantic, at least for some forms of synesthesia, the development of synesthetic associations has to be related to the development of person’s semantic knowledge base. The process of learning, education and acquiring knowledge about the surrounding world should in some instances give rise to synesthetic associations. Insights into how a child’s learning is associated with synesthesia can be identified from the characteristics of the most common types of synesthesia. A pertinent question is the following: why are the letters, numbers and the time-units the most common inducers of synesthesia (Simner et al., 2006; Cytowic and Eagleman, 2009). We propose that these kinds of inducers are frequently the first truly abstract concepts that a child has to learn, at least in Western civilization.

The following set of hypotheses can be formulated: until a child begins to learn letters, numbers and days of the week, he or she is mostly faced with very concrete objects, such as particular toys, food, items of clothing, etc. These objects have very specific perceptual representations, which can be memorized and hence, are relatively easy to manipulate mentally. But then comes the time when a child must learn abstract concepts. One cannot see Tuesday or Sunday. There are no basic and straightforward perceptual memories to be formed for days of the week. Similarly, numbers (number names) are not just words but they have an abstract meaning of quantity that can apply to any objects (Teddy bears, bananas or shoes). This is one step more abstract than what the child was used to because none of the perceptually well-supported images can be used as an identifier of numerosity (there could be three Teddy bears, three bananas and three shoes but nothing can explicitly visualize the meaning of number three). Likewise, one and the same letter can stand for many different words and thus, for many different objects.

As kids, we all have to find a way to acquire those abstract concepts. Synesthetes may have an advantage there. They can assign to those concepts something concrete, perceptual-like, that will always be present irrespective of the actual objects in the external world. They can assign a color to the concept, or a spatial position, or both. That way, they can accelerate their mental manipulations of those concepts: recall them quicker and more accurately, determine the relationships between them, and so on.

Synesthesia then becomes a tool for accelerating mental operations with cognitively demanding i.e., abstract, contents. This mostly aids the minds that have not yet been elaborated and have not developed the advanced, adult-like, abstract mechanisms of thinking. That is, this helps primarily the child’s mind. And this explains why the most frequent forms of synesthesia involve letters, numbers and time units. Longitudinal studies of synesthesia starting in early childhood and reaching into the adult years will likely be of great help as a way to shed light on these processes (Simner and Bain, 2013; Meier et al., 2014).

## Discussion

Generally, perceptual experiences are richer and more fine-grained than our conceptual apparatus, i.e., they contain more distinct dimensions and perceptible positions on each dimension. Non-conceptual contents of perceptual states are more concrete and specific than are general conceptual contents of cognitive states (Evans, 1982; Heck, 2000; Peacocke, 2001a,b). Thus, our ability to discriminate perceptually particular sensory values exceeds the ability to conceptualize them. For example, we can notice the difference between specific hues of blue, such as Blue No. 37 and Blue No. 38, when presented simultaneously. Nevertheless those hues remain ineffable nuances to most of us (Raffman, 1995; Metzinger, 2009).

What we learn from studying the semantic aspects of synesthesia is that for synesthetes, unlike for non-synesthetes, even abstract concepts receive some of these fine-grained qualities to be specifically seen, heard, smelt, tasted, felt, or otherwise experienced. For example, digits have certain, very distinct, personalities, musical notes are precisely colored, words contain specific flavors or colors, time units may be positioned at particular locations in space, and emotions can similarly smell and sound distinctively (Baron-Cohen et al., 1987; Grossenbacher, 1997; Steven and Blakemore, 2004; Simner et al., 2006, 2009; Ward et al., 2006; Sinha, 2009; Amin et al., 2011; Figure 2).

An analogous ability in the general non-synesthetic population is discussed in philosophical debates such as that of whether there are high-level properties in the content of perception (Siegel, 2006; Bayne, 2009). Those who think that there are such properties in the content of perception hold that high-level properties enter into phenomenal contents. Another debate pertains to *cognitive phenomenology*, according to which phenomenology extends beyond the sensory domain (Pitt, 2004; Bayne and Montague, 2011). This means that phenomenal consciousness cannot be reduced to non-conceptual contents of perceptual states representing sensory properties. Phenomenal experiences also involve various cognitive states including high-level conceptual contents. Apparently, not only non-conceptual attributes determine the character of our phenomenology; conceptual representations also affect our phenomenal experiences. Thus, both perceptual and cognitive states are causally and explanatorily relevant in elucidating how we experience the world because they both exhibit their own phenomenal characters**—***something it is like to be* in such a state for the subject (Strawson, 1994; Chalmers, 1996; Montague and Bayne, 2011; cf. Nagel, 1974), e.g., both seeing an object of deep red color and understanding a complicated mathematical formula are associated with certain phenomenal qualities.

Here we have shown that synesthesia combines a rich diversity of phenomenal contents—a multitude of experiential levels that are primarily driven by: sensory, motor, bodily, emotional or conceptual systems. Consequently, the full combined experience of synesthesia exhibits a holistic epistemic unity—an overall integration of experiences driven by different mental faculties, cognitive and sensory, which are then bound into a coherently unified conscious experience (Mroczko et al., 2009; Mroczko-Wąsowicz, 2013). In this way, synesthesia transgresses the boundaries between perception and cognition more than any other mental phenomenon—i.e., between capacities traditionally considered as two independently operating domains (Mroczko-Wąsowicz and Werning, 2012).

The cross-domain experiences should not be seen only as an exception, as if it would only be the case for the extraordinary phenomenon of synesthesia. Rather, they should be seen as a general rule applicable also to non-synesthetic practices. If this premise is accepted, synesthesia may be understood as making use of mechanisms similar to those present in ordinary perception and imagery (Ward et al., 2007). The reason why a synesthete takes an extra step in creating a unique synesthetic experience may lie in the nature of the individual semantic network of concepts s/he is deploying. As Jürgens and Nikolić (2014) proposed, the semantic structure of each inducing category (e.g., a letter of an alphabet) may be richer for a synesthete by one attribute than for a non-synesthete. Thus, besides the size, shape, or position in the alphabet, or an associated sound, a synesthete would have one more attribute for each grapheme—its color.

In general, we propose that perception and conceptual knowledge are intimately linked and should be investigated together as a unified research problem. By doing so, more progress may be made in understanding human cognition—both synesthetic and non-synesthetic. Thus, lessons learned from synesthesia may be extended into the “ordinary world” of perception and help us develop an integrative approach. This approach would recognize that the boundary between cognition and perception is elusive: perception is more sophisticated than traditionally assumed, rising up to the “high-level”. And similarly, “low-level” cognitive operations are fundamentally grounded in our perceptual capabilities (Schyns et al., 1998; Goldstone et al., 2010; Goldstone and Hendrickson, 2010).

Much evidence exists that perception affects cognition such as belief acquisition and formation of concepts (Prinz and Barsalou, 2000; Goldstone et al., 2008; Barsalou, 2009, 2012). This supports the hypothesis of *concept empiricism*, according to which conceptual representations and abstract conceptual knowledge are perceptually based. The underlying operations involve re-activation of perceptual and motor representations (Goldstone and Barsalou, 1998; Barsalou, 1999; Prinz, 2002, 2005). Conceptual contents affect not only the phenomenal character of cognitive states (Montague and Bayne, 2011), but they can equally so inform and influence the phenomenal character of perceptual states; in other words they can cognitively penetrate our perception (Macpherson, 2012). For example, changes in the conceptual content of the inducing stimulus (e.g., the mood or new knowledge about the stimulus) modify perception of the synesthetic concurrent.

This relationship between perception and conception is an integral part of the practopoietic theory and specifically, of the presumed mechanisms of *anapoiesis* responsible for extraction of knowledge from long-term to working memory (Nikolić, 2014). In this system, the process of perception and the process of conceptualization of the stimulus rely on the same underlying mechanisms. That is, there is little difference between perceiving a situation or an object (e.g., a chair) and conceptualizing that object, including the most general aspects such as the means of interacting with that object (e.g., knowing what movements are needed in order to sit on a given chair).

Also, our proposal, that semantic mechanisms may be responsible for generating some forms of synesthesia (i.e., an ideasthesia) is compatible with *cognitive penetrability of perception*, a recent interdisciplinary approach based on the presumption that there are various ways in which conscious perception can be modified by cognition–i.e., by thoughts, beliefs, desires, judgments, intentions, moods, emotions, expectations, knowledge, previous experiences and memories (Frith and Dolan, 1997; Bar, 2003; Raftopoulos, 2005, 2009; Vuilleumier and Driver, 2007; Stokes, 2012; Wu, 2013; Vetter and Newen, 2014). In other words, conceptual contents of higher cognitive states not only have causal influence on the contents of perception, but they are explanatorily relevant when trying to account for the processing of perceptual systems. Similarly relevant are the semantic aspects of inducers for explaining the induction of concurrents in synesthesia. A full understanding of how the mind works requires considering the tight relations holding among the cognitive and perceptual domains and their mutual impact.

## Conflict of interest statement

The authors declare that the research was conducted in the absence of any commercial or financial relationships that could be construed as a potential conflict of interest.
